# Artificial Intelligence to Aid Glaucoma Diagnosis and Monitoring: State of the Art and New Directions

**DOI:** 10.3390/photonics9110810

**Published:** 2022-10-28

**Authors:** Roberto Nunez, Alon Harris, Omar Ibrahim, James Keller, Christopher K. Wikle, Erin Robinson, Ryan Zukerman, Brent Siesky, Alice Verticchio, Lucas Rowe, Giovanna Guidoboni

**Affiliations:** 1 Department of Electrical Engineering and Computer Science, University of Missouri, Columbia, MO 65211, USA; 2 Department of Ophthalmology, Icahn School of Medicine at Mt. Sinai, New York, NY 10029, USA; 3 Department of Electrical Engineering, Tikrit University, Tikrit P.O. Box 42, Iraq; 4 Department of Statistic, University of Missouri, Columbia, MO 65211, USA; 5 Department of Social Work, University of Missouri, Columbia, MO 65211, USA; 6 Department of Ophthalmology, Edward S. Harkness Eye Institute, Columbia University Irving Medical Center, New York-Presbyterian Hospital, New York, NY 10034, USA; 7 Department of Ophthalmology, Indiana University School of Medicine, Indianapolis, IN 46202, USA; 8 Department of Mathematics, University of Missouri, Columbia, MO 65211, USA

**Keywords:** artificial intelligence, glaucoma, mathematical modeling, statistical modeling, glaucoma progression, glaucoma diagnosis

## Abstract

Recent developments in the use of artificial intelligence in the diagnosis and monitoring of glaucoma are discussed. To set the context and fix terminology, a brief historic overview of artificial intelligence is provided, along with some fundamentals of statistical modeling. Next, recent applications of artificial intelligence techniques in glaucoma diagnosis and the monitoring of glaucoma progression are reviewed, including the classification of visual field images and the detection of glaucomatous change in retinal nerve fiber layer thickness. Current challenges in the direct application of artificial intelligence to further our understating of this disease are also outlined. The article also discusses how the combined use of mathematical modeling and artificial intelligence may help to address these challenges, along with stronger communication between data scientists and clinicians.

## 1. Introduction

Artificial intelligence (AI) widely refers to the ability of digital machines or computers to accomplish tasks with minimal human involvement. AI has been employed throughout many industries, including finance, marketing, and travel, and has gained traction more recently in medicine. AI-assisted medical screening, diagnosis, and treatment is now being used to allow healthcare providers to deliver care to patients more effectively and precisely. Historically, ophthalmology has been a very technology-driven medical specialty, and AI is now being implemented to assist in the diagnosis, monitoring of progression, and treatment of ophthalmologic conditions, most notably glaucoma.

The growth of AI applications in ophthalmology has risen sharply over the past two decades, in conjunction with a wealth of diverse imaging data [1]. As a multifactorial disease, glaucoma is uniquely suited for AI applications, where interpreting vast amounts of data generated from the heavily technology-focused diagnostic platforms requires dynamic learning and non-statistical approaches. As evidence of growth in the field, at the 2022 annual meeting of the Association for Research in Vision and Ophthalmology (ARVO), over ten paper sessions were devoted to the study of artificial intelligence in ophthalmology.

Glaucoma is one of the leading causes of irreversible blindness in the world. Worldwide in 2010, approximately 60 million people suffered from the disease, with estimated increases to 76 million in 2020 and 112 million by 2040 [2]. Intraocular pressure (IOP) has been considered the most significant risk factor for the development and progression of open-angle glaucoma (OAG) [3]. However, many patients develop glaucoma and experience disease progression despite IOP measurements within normal ranges [4]. Risk factors shown to be involved in the onset and progression of OAG include age, race, gender, blood pressure (BP), cerebrospinal fluid pressure, systemic vascular dysregulation, central corneal thickness (CCT), myopia, and diabetes mellitus, among others [1].

Glaucoma is a truly multifactorial disease with highly individual risk factors. The progression of the disease is often slow and subtle, resulting in irreversible vision loss well before diagnosis. Thus, early identification of glaucomatous change and optimal initiation of treatment are crucial in preventing disease progression.

Given its capability of processing large and complex datasets, AI provides a natural complement to the technologies that are available to clinicians and could greatly influence how the disease is diagnosed and managed early in its course. Furthermore, given the strongly multifactorial nature of glaucoma and the limitations of the current technologies in assessing all of its risk factors, the application of AI to glaucoma calls for the development of innovative AI approaches that may prove beneficial for many other multifactorial diseases.

The present work aims to provide a broad overview of the application of AI to the study of glaucoma. Section 2 introduces AI from a historical perspective and examines how its meaning has evolved over time to embrace a wide variety of different computer methods for analyzing data, with and without human supervision. Next, AI methods that aid the diagnosis of glaucoma and the monitoring of its progression are reviewed in Sections 3 and 4. Sections 5 and 6 illustrate how AI methods can be complemented with methods based on statistical and physics-driven modeling. Finally, challenges and new directions are discussed in Section 7.

## 2. What Is AI?

What is Artificial Intelligence? It depends on whom and when you ask. In 1955, John McCarthy coined the term “Artificial Intelligence” in a proposal with Marvin Minsky, Nathaniel Rochester, and Claude E. Shannon for the now famous Dartmouth conference in the summer of 1956 [5]. He defined AI as “the science and engineering of making intelligent machines”. McCarthy et al. conjectured “that every aspect of learning or any other feature of intelligence can in principle be so precisely described that a machine can be made to simulate it.” While the proposal went on to discuss natural language processing, neuron networks, the theory of computation, abstraction, and creativity, early work focused on the mechanisms of rational thought that are embodied in binary symbolic logic [6]. Realizations were instantiated by expert systems that contained symbolic rules and facts and used the principles of first-order binary (crisp) logic to produce deductions [7,8]. To address issues of uncertainty, probabilities were somehow assigned to rules and were handled in parallel to the inference procedures. This emphasis on modeling rational thought remains a cornerstone of AI; it was extended to more closely model human thought via fuzzy logic, based on the theory of fuzzy sets that was pioneered by Lotfi Zadeh [9,10]. The basic propositions are modeled by fuzzy sets that are meant to reflect the vagueness and imprecision inherent in human linguistic expressions.

Fuzzy logic is one way to move from the classical AI focus on symbolic logic as the model of rational thought. The inference process itself uses functions and numbers instead of crisp symbols. Many other techniques, under the general term of machine learning, also teach and develop computational machines that perform tasks associated with human intelligence, such as decision making, pattern recognition, planning, adapting, and even generalizing. Almost all of these techniques utilize numeric features and calculations. They can either be supervised (based on labeled training data) or unsupervised (clustering and other data analytics). All neural network models, including those under the umbrella of deep learning (usually based on huge, labeled data training datasets) fit into this category [11,12]. (Actually, the expression computational intelligence (CI) was coined to distinguish between the symbolic logic of early AI and those computational models, particularly neural networks, fuzzy systems, and evolutionary computation [13]). A broader definition of AI includes all of these techniques, and in this paper, we adopt this more general understanding as our definition of AI

Finally, for AI assistance to be useful, understood, and believed by a human expert, it should be transparent (the model is actually described by humans), interpretable (after training, the human can view and understand the model itself), and/or explainable (usually interpreted to mean that the learned model will produce statements or visualizations to demonstrate how it made decisions). Explainable AI (XAI) is thought of as the third wave of artificial intelligence [14,15].

It is important to bear in mind that AI predictions are based on data and, consequently, they can only be as good as the data they are built upon. Thus, to make AI predictions more effective, it is essential to have (i) large datasets, so that the algorithms yield accurate results, and (ii) relevant features, so that the outcomes can be interpreted in meaningful ways. Sections 3 and  4 will be concerned with large datasets, while in Section 5 we will discuss some recent efforts to address relevant features. Moreover, there are inherent risks of both intentional and unintentional bias associated to the use of data and AI to make predictions. These risks should be understood and addressed when utilizing AI to further our understanding of a given field. We refer the reader to [16] for a systematic account of bias in AI models.

## 3. AI and Statistical Modeling

Many statistical learning and machine learning paradigms can be considered as AI models. From a supervised modeling perspective, the Bayesian inferential paradigm is the most natural for AI applications. Such methods are grounded in formal probability theory (see, e.g., [17]). In its simplest form, assume we have a collection of observations given by Y and we have data generating model that depends on a collection of parameters, P. We specify the data generating model, called the “likelihood”, by the distribution [Y|P], meaning the distribution of Y given the parameters P. Bayesian methodology then assumes that the parameters should be considered as random variables, and one must specify a “prior” probability distribution for them, say [P]. One is interested in making an inference about P, given the data Y, and we can obtain this distribution (known as the “posterior” distribution) using Bayes Rule: [P|Y] ∝ [Y|P][P]. Critically, this is only a proportionality (hence, the symbol ∝), and we must normalize this distribution by [Y] (i.e., by marginalizing P from [Y|P][P]). Outside of simple problems, this normalizing constant cannot be obtained analytically, and one must use computational methods to obtain it. In the mid-1990s, it was realized that Markov chain Monte Carlo methods could be used for these revolutionized Bayesian statistics and set the stage for much more complex hierarchical (multi-level) Bayesian models that could accommodate much more complex data and underlying generating processes, such as Gaussian processes (GPs) or Markov random fields (see, e.g., the summaries in [18,19]). More recently, approximate computational methods that admit greater scalability, such as variational Bayesian methods, have been developed to accommodate Bayesian inference for very large data sets. Most AI applications of Bayesian methodology are based either on Bayesian hierarchical models (BHMs), GPs, or variational Bayesian methods (e.g., variational autoencoders, or VAEs). We discuss these approaches below, in the context of glaucoma.

The advantage of the BHM framework is that it is a multi-level (“deep”) probabilistic modeling framework that relies on a series of telescoping conditional distributions that are all formally linked. As outlined in [19], this framework is ideal for fusing multiple data sets, accommodating complex spatial and temporal dependencies, accounting for parameter uncertainty directly, and incorporating a prior information if it is available. In the context of glaucoma, these models have been used for over a decade (see, e.g., [20–25]). The common theme in these papers was dealing with complex dependence, associated either with longitudinal study designs, spatial (image) effects, or temporal changes (disease progression). Perhaps the best illustration of these complex BHM applications to glaucoma modeling is [22], which considered a BHM functional model with data consisting of correlated functions on the spherical scleral surface. That study included nonparametric age effects, multi-level random effects to account for within-subject dependence, and functional growth terms that captured temporal dependence across IOPs that varied on the scleral surface. Importantly, all of these components were integrated into a coherent Bayesian probability model that allowed for complex dependencies and uncertainty quantification.

Gaussian processes (GPs) have long been used to model spatial processes (e.g., optimal interpolation of missing observations, as summarized in [19]), and more recently used in the context of flexible regression modeling in machine learning (see, e.g., [26]). The advantage of these methods is that they can model flexible features and accommodate uncertainty quantification (see, e.g., [27]). Although GPs can be implemented from frequentist or Bayesian paradigms, the Bayesian approach is most common, due to the desire to obtain formal uncertainty quantification in the predictions and parameter estimations that are associated with GPs. For example, [28] proposed an approach for retinal blood vessel tracking and diameter estimation by modeling the curvature and the diameter of blood vessels as GPs. More generally, GPs are strongly connected to deep neural networks. For example, ref. [29] showed that there is an exact equivalence between infinitely wide deep networks and GPs. This provides the advantage of full uncertainty quantification with the GP formulation, which is not available for traditional deep neural models.

The third area of significant intersection between statistical modeling and AI is via variational Bayesian inference. This is an approximate Bayesian inference procedure that provides much more scalable implementations in complex modeling than that of traditional Bayesian computation (e.g., MCMC). For example, variational autoencoders are a type of generative AI model (in the same class as generative adversarial networks) that have traditionally been used to generate realistic spatial structures in images. They utilize a combination of deep neural models to learn (random) latent variable structures that serve to generate complex dependencies within a Bayesian statistical modeling framework, implemented with an approximate (but scalable) variational procedure. For example, [30] used VAEs in a spatio-temporal context to model the spatial maps associated with visual field tests in a longitudinal study that monitored signs of glaucomatous progression. An alternative use of such methods is to increase power in image-based studies by the realistic construction of augmented data (see, e.g., [31]).

## 4. Glaucoma Diagnosis

AI and deep machine learning offer the ability to augment the identification of risk factors and biomarkers to aid in the early diagnosis and classification of glaucoma. Glaucoma screening is particularly important, as the disease is asymptomatic in its course. As the diagnosis and monitoring of many ocular diseases rely upon pattern recognition of ophthalmic imaging, these emerging technologies have the potential to outperform current manual methods of interpretation. Currently, glaucoma is diagnosed by an ophthalmologist’s performance of a comprehensive ophthalmic examination and diagnostic testing. The American Academy of Ophthalmology cites two forms of damage (structural and functional) in its definition of OAG [32]. The structural damage refers to the retinal nerve fiber layer (RNFL) or to optic disc structural abnormalities (such as decreased RNFL thickness or an increased cup-to-disc ratio) that can be assessed with multiple non-invasive imaging, such as that provided by Heidelberg retinal tomography (HRT) or optical coherence tomography (OCT). Functional damage encompasses visual field (VF) defects that are reliable and reproducible without an alternative explanation of cause and are assessed by VF testing. IOP measurements are an important part of the ophthalmic examination, though IOP elevations alone are not sufficient to diagnose OAG. Importantly, other testing approaches that are crucial for glaucoma diagnosis include the evaluation CCT, a parameter that can influence IOP measurement, and gonioscopy. It is important to monitor both structural parameters and functional parameters regularly for the progression of the disease over time.

One recent clinical trial compared the accuracy of a deep convolutional neural network (CNN) with that of resident ophthalmologists, attending ophthalmologists, glaucoma experts, and traditional guidelines (Advanced Glaucoma Intervention Study (AGIS) score and Glaucoma Staging System 2 of Brusini (GSS2)) in the differentiation of glaucoma from non-glaucoma VFs. In that study, the diagnostic criteria for glaucoma were similar to those of the UKGTS study. In addition, patients with glaucomatous damage to the optic nerve head (ONH) and reproducible glaucomatous VF defects were included in the study. ONH damage was defined as C/D ratio ≥ 0.7, thinning of RNFL, or both, without a retinal or neurological cause of VF loss. A glaucomatous VF defect was defined as a reproducible reduction of sensitivity, compared with the normative database in reliable tests at (1) two or more contiguous locations with *p* < 0.01 loss or more, or (2) three or more contiguous locations with *p* < 0.05 loss or more. The CNN model was trained with a set of 3712 VF images; the convolutional neural network achieved higher accuracy in the differentiation of glaucoma and non-glaucoma, compared with that of human ophthalmologists, in a set of 300 VF images for validation [33]. That trial emphasized the opportunity that can be provided by deep machine learning in support of diagnosing glaucoma patients.

Medeiros et al. [34] investigated a new approach for the objective quantification of glaucomatous damage by training a deep learning convolutional neural network to assess fundus images and predict spectral-domain (SD) OCT average RNFL thickness. Glaucoma diagnosis was defined on the basis of the presence of glaucomatous repeatable visual field loss in SAP (pattern standard deviation [PSD] < 5% or glaucoma hemifield test outside normal limits) and signs of glaucomatous optic neuropathy, based on records of slit-lamp fundus examination. Patients were defined as individuals suspected of having glaucoma if they had a history of elevated intraocular pressure, suspicious appearance of the optic disc on slit-lamp fundus examination, or other risk factors for the disease. On the other hand, healthy subjects were defined as those with a normal optic disc appearance on slit-lamp fundus examination in both eyes, no presence of elevated intraocular pressure, and normal SAP results. The cross-sectional study included 32,820 pairs of optic disc images, 2312 SD OCT RNFL scans, and evaluated correlation and agreement between predicted and actual SD OCT thickness, as well as on the ability to differentiate between eyes with glaucomatous VF loss and healthy eyes. That study found that there was a very strong correlation between deep learning algorithm-predicted and observed SD OCT thickness, in addition to a strong similarity between the ability of the deep learning algorithm and the actual SD OCT RNFL measurements in distinguishing between glaucomatous and healthy eyes. That study introduced a novel deep learning approach to read fundus images and potentially diagnose and stage glaucomatous damage without the requirement of human labeling of a reference training set.

Similarly, a study by Jammal et al. [35] used a machine-to-machine deep learning (M2M DL) algorithm to compare its efficacy to that of glaucoma specialists in the detection of glaucomatous changes in RNFL thickness and the cup-to-disc ratio. The presence of reproducible glaucomatous defects was defined by using SAP as the reference outcome to fairly compare the performance of the human graders and the M2M DL algorithm in detecting glaucoma. In case of disagreement between the graders, four reliable SAP tests (two preceding and two following the photo-matched SAP) were extracted from the repository for each eye and manually reviewed by two graders who reached a compromise agreement. Furthermore, eyes were marked with repeatable glaucomatous field defects if they had clear patterns of glaucomatous visual field loss (e.g., arcuate scotomas or nasal steps) that were consistently present throughout the visual field series. Functional loss on SAP was the main reference for a glaucoma diagnosis. The classification in that study targeted, primarily, discrimination between eyes with and without a repeatable glaucomatous visual field loss. It is worth mentioning that it is possible that some eyes with glaucoma may have been included in the normal visual field group, due to the lack of perfect reference for glaucoma diagnosis. The (M2M DL) algorithm was applied to a subset of 490 fundus photos that were graded by two glaucoma experts for the probability of glaucomatous optic neuropathy and estimates of cup-to-disc ratios. The estimates provided by the experts and the deep learning algorithm were compared to Spearman correlations with standard automated perimetry, with the algorithm performing significantly higher than the human graders. The results from this study suggested that deep learning algorithms may provide a reliable aid for glaucoma experts in the identification of retinal nerve fiber layer thinning and glaucomatous optic neuropathy when screening for glaucoma.

An abstract presented to the American Glaucoma Society (AGS) by Thompson et al. [36] investigated the use of a deep learning algorithm, free of the conventional segmentation of the RNFL, to assess glaucomatous damage on the entire circle B-scan image from SD OCT. All eyes in the study had baseline OD photographs and were monitored over time with SDOCT RNFL thickness measurements. The estimation of the rates of change in global RNFL thickness over time was achieved using linear mixed models. The segmentation-free deep learning algorithm was found to perform significantly better than conventional RNFL thickness parameters in the diagnosis of glaucoma. The use of this algorithm may provide clinicians with a more reliable tool to detect glaucomatous change than the error-susceptible segmentation of RNFL.

Early diagnosis of glaucoma is crucial for a better treatment outcome. Medical practitioners have proposed different approaches for early diagnosis and these criteria primarily focus on or around the Optic Disc (OD) region. Accurately calculating the position, center, and size of the OD can significantly help in further automated analysis of the image modality. In [37], a deep convolutional neural network was proposed in a two-step framework, both to detect the optic disc on fundus images and to classify them as glaucomatous or healthy. The neural network was tested on seven publicly available datasets for disc identification and the ORIGA-light database for glaucoma classification. The ORIGA-light database is the largest publicly available dataset, with both glaucoma and healthy images. Given that the ground truth for glaucoma diagnosis used in the various datasets was not available to the authors, they devised their own semi-automated ground truth generation method, using a rule-based algorithm. The results of that study found that the neural network reached a new record level of accuracy in the identification of optic discs, reaching 100% accuracy in four of the image sets. The neural network revealed a 2.7% relative improvement in glaucoma classification, compared with previously obtained results on the ORIGA-light dataset.

Kucur et al. [38] developed a convolutional neural network to investigate its efficacy in discriminating VFs between healthy and early glaucomatous eyes. Two VF sets from the OCTOPUS 101 G1 program and the Humphrey Field Analyzer 24-2 pattern were subdivided into control and early-glaucoma groups and used to train the convolutional neural network, with saliency maps generated to highlight which regions of the VFs contributed the most to the model’s classification. For the first dataset, healthy eyes were selected if they had no optic nerve head damage and had reliable and reproducible normal OCTOPUS G1 VF results, an MD < 2.0 dB, an LV < 6.0 dB, with no significantly decreased test point sensitivity values and intraocular pressure consistently below 21 mm Hg. The under-treatment OHT eyes were those with a normal VF with MD < 2.0 dB and LV < 6.0 dB and a normal optic nerve head. In addition, the under-treatment perimetric glaucoma eyes had definite glaucomatous neuroretinal rim loss, and reliable and reproducible VF defects that are typical with glaucoma. Finally, the under-treatment perimetric glaucoma eyes were those with glaucomatous neuroretinal rim loss reliable and reproducible normal OCTOPUS G1 VF results, an MD < 2.0 dB, and an LV < 6.0 dB. For the second dataset, both eyes of the subjects were tested using a white-on-white 24–2 test pattern with the fullthreshold algorithm during a span of 5 to 10 years. The model was then tested for average precision and compared to mean defect, square root of loss variance, their combination, and a non-convolutional neural network. Their results revealed that their convolutional neural network demonstrated generally superior performance in comparison with the other methods, and the computed saliency maps provided clinically relevant information on regional VF loss for justification of the model’s classification.

Ahn et al. [39] similarly trained a deep learning model to diagnose both early and advanced glaucoma using fundus photography. The normal patients were those with normal findings on red-free RNFL photography (Vx-10; Kowa Optimed, Inc., Tokyo, Japan), OCT (Cirrus HD-OCT, Carl Zeiss Meditec Inc., Dublin, CA, USA), and visual field testing (Humphrey 740 visual field analyzer, Carl Zeiss Meditec Inc., Dublin, CA, USA). The inclusion criteria of the glaucoma patients were as follows: typical glaucomatous visual field defects, and/or a bundle of defects of RNFLs on HD-OCT, and/or a bundle of defects of RNFLs on red-free RNFL photography. Fundus photos of 786 normal controls, 467 advanced glaucoma patients, and 289 early glaucoma patients were divided into training, validation, and testing sets to construct both a simple logistic classification and a convolutional neural network, in addition to further tuning a pre-trained GoogleNet Inception v3 model. The new convolutional neural network was found to perform better than the other two models in detecting both early and advanced glaucoma from fundus photographs alone.

## 5. Glaucoma Progression

While AI is emerging as a tool for clinicians in augmenting data collection and informing clinical decision-making surrounding glaucoma diagnosis and treatment, the most important future role of AI may be providing a better understanding and monitoring of glaucoma disease progression. Traditionally, glaucoma progression has been defined by two classifications: structural progression and functional progression, which are generally understood to occur in both an independent and dependent manner [40].

Structural progression is defined by measurements of the neuroretinal rim area, RNFL thickness, and the cup-to-disc ratio expressed as units of change per year [41]. Functional progression is defined by VF testing and analysis of VF-derived indices, such as mean deviation and the VF index (VFI), which are both expressed linearly. In order to standardize these functional measurements, scoring systems, such as the AGIS and Collaborative Initial Glaucoma Treatment Study (CIGTS) scores, have been developed [42,43]. However, many studies, such as the ones we survey in this article, have not employed standard scoring systems, but rather concerned themselves with the analysis and prediction of clinical markers, without deducing a progression status from them.

Archetypal analysis, an AI algorithm, was used to determine central VF patterns and perform longitudinal analyses to investigate the development of central VF defects in specific vulnerability zones in end-stage glaucoma patients. The algorithm was applied to data curated from the Glaucoma Research Network. A total of 2912 reliable 10-2 VFs of 1103 eyes from 1010 patients, measured after end-stage 24-2 VFs with a mean deviation (MD) of −22 dB or less, were included in the analysis. The algorithm helped to reveal that initial central VF loss in end-stage glaucoma is likely to be nasal and that one specific pattern of nasal loss is more likely to progress to total loss [44].

An AGS abstract provided by Dharia et al. [45] used a DL algorithm as a prediction tool for the timing of interventions in the treatment of glaucoma. Their study employed a convolutional neural network (CNN) using a three-fold cross-validation scheme to calculate the probability of intervention after the fourth visit, with data on the ages, VFs, and IOPs of patients who underwent laser trabeculoplasty or glaucoma surgical interventions. The CNNs revealed that IOP can act as a sensitive indicator for the timing of interventions and VF can act as a sensitive indicator for determining when intervention is not necessary. The use of all three predictors in age, IOP, and VF displayed high sensitivity and specificity in predicting the timing of glaucoma procedural interventions.

Rule-based techniques for assessing glaucoma progression from VFs only are conflicting and have tradeoffs. A convolutional long short-term memory (LSTM) neural network is used to study glaucoma progression on a longitudinal dataset of merged VF and clinical data. The dataset used in the study has 11,242 eyes where each sample has four or more VF results and corresponding baseline clinical data (cup-to-disc ratio, CCT, and IOP). Three glaucoma progression algorithms (VF index slope, mean deviation slope, and pointwise linear regression) were employed to define eyes as progressing or stable. Two LSTM algorithms were tested: one was trained on VF data, and the other was trained on both VF and clinical data. The convolutional LSTM network demonstrated 91% to 93% accuracy, compared with the different conventional glaucoma progression algorithms. The authors concluded that the model that was trained on both VF and clinical data showed better diagnostic ability than a model trained on VF results only, because combining both VF results and the clinical data improved the model’s ability to assess glaucoma progression and better reflected the way clinicians manage data when managing glaucoma [46].

Park et al. [47] built a VF prediction algorithm using recurrent neural networks (RNN). They used the conventional pointwise ordinary linear regression (OLR) technique to evaluate the performance of the proposed approach. A dataset of 1408 eyes was used in the training phase and another dataset with 281 eyes was used in the testing phase. The input to the constructed RNN consisted of five consecutive VF tests, and a sixth VF test was compared with the output of the RNN. That study showed that the overall prediction performance of RNN was significantly better than that of OLR, with less pointwise prediction in most areas that are known to be vulnerable to glaucomatous damage. The authors conceded that RNN is more robust and reliable with respect to worsening in the VF examination.

In many studies, a scalar representation for RNFL has been used in predicting glaucoma progression. That method discards useful spatial information that could potentially be of relevance. Nagesh et al. [48] proposed a spatio-temporal approach to predict longitudinal glaucoma measurements, using a Continuous-Time Hidden Markov Model (CT-HMM). Two common glaucoma biomarkers (RNFL thickness for structure and VFI for function) were used in that study. The authors proposed a technique to incorporate the spatiotemporal RNFL thickness measurements obtained from a sequence of OCT images into a longitudinal progression model. Then, CT-HMM was used to jointly model the change in RNFL thickness via VFI and predict future measurements. The authors achieved a decrease in mean absolute error of 74% for spatial RNFL thickness encoding, in comparison with prior studies, which used the average RNFL thickness. Such a model can be useful in predicting the spatial location and intensity of tissue degeneration.

Wen et al. [49] investigated the use of deep learning in forecasting future 24–2 Humphrey VF (HVFs). A dataset with 32,443 24–2 HVFs was used in the study. Ten-fold cross validation with a held-out test set was used to train a deep learning neural network capable of generating a point-wise VF prediction. The authors concluded that deep learning showed the ability not only to learn spatio-temporal HVF changes but also to generate predictions for future HVFs up to 5.5 years, given only a single HVF.

Garway-Heath et al. [50] proposed an extensive study to compare statistical methods that used VF and OCT with methods that used VF only. The aim of their study was to test whether the combination of VF and OCT led to more rapid identification of glaucoma progression and shorter clinical trials. The reference progression detection method was based on Guided Progression Analysis (GPA) Software (Carl Zeiss Meditec Inc., Dublin, CA, USA). The study revealed that combining VF and OCT data had a higher hit rate and identified progression more quickly than the reference and other VF-only methods. The method combining VF and OCT data also produced more accurate estimates of the progression rate but did not increase treatment-effect statistical significance.

## 6. AI in Ophthalmology: Current Challenges and Future Directions

Saeed et al. [51] conducted a study to determine the agreement of six established VF progression algorithms in a large dataset of VFs from multiple institutions. A subset of 90,713 VFs from 13,156 eyes of 8499 patients was used in the experiment. Each eye was assigned to be stable or progressing, using each of the six measures. Cohen’s k coefficient was employed to test the agreement between the individual measures. In addition, they used bivariate and multivariate analyses to determine predictors of discordance. The results revealed poor-to-moderate agreement between individual algorithms, when compared directly. That study demonstrated that existing VF algorithms have limited agreement and that agreement varies with clinical parameters, including institutional parameters. These issues highlight the challenges in the clinical use and application of progression algorithms and the application of big-data results to individual practices.

The soundness of an AI model heavily relies on the quality of the data on which it is trained. In the study, diagnosis, and monitoring of glaucoma, there are two main challenges regarding the collection and processing of data that may hinder the use of AI in the field. Below, we discuss these challenges and some of the recent efforts to overcome them.

The unavailability of potentially key data poses a challenge to the use of AI in glaucoma diagnosis and management. Standard glaucoma screening consists of a complete ophthalmic examination that includes, an assessment of IOP, CCT, gonioscopy, an assessment of visual function via VF testing, an assessment of structural damage at the level of the optic nerve, and RNFL via multiple imaging devices [32]. However, hemodynamic variables not considered in these screenings may carry relevant information regarding the onset and development of glaucoma. Even though the vascular status of selected tissues and blood vessels in the eye can be assessed via several non-invasive imaging techniques, such as OCT angiography (OCTA), Heidelberg retinal flowmetry (HRF), color Doppler imaging (CDI), and retinal oximetry, these instruments are often only available in clinical research centers. Moreover, there is currently no technology that is widely available for measuring hemodynamic variables pertaining to the venous side of the circulation. Accordingly, the potential influence of hemodynamics in glaucoma, especially in the veins, may not be discernible from the available data, leading to biases in AI models.

**Figure 1.**
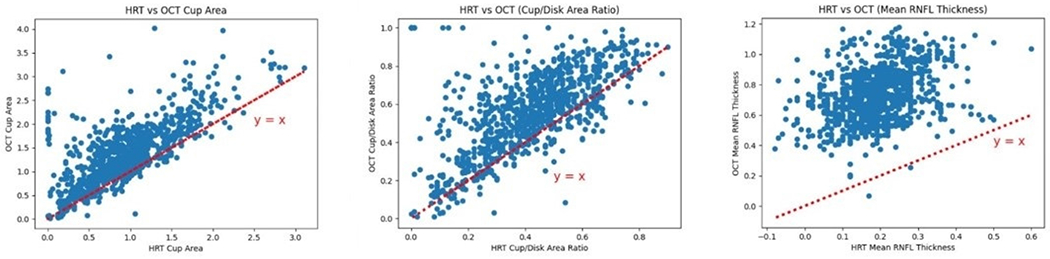
Comparison of Heidelberg retinal tomography (HRT)- vs. optical coherence tomography (OCT)-derived parameters: (**left**) cup area, (**center**) cup to disk area ratio, (**right**) mean retinal nerve fiber layer (RNFL) thickness.

Even when instruments are readily available, clinical measurements pertaining to the same ocular parameters are not necessarily consistent when performed with different instruments, thereby leading to what is known as non-commensurate data. For example, the RNFL thickness is an important marker of glaucomatous damage, with RNFL thinning being related to the loss of retinal ganglion cells [52–56]. Both OCT and HRT provide estimates of RNFL thickness, but they do so by means of different physical principles. OCT performs interferometry to discriminate between tissues with different optical properties in the retina and evaluates RNFL from a signal produced within the retinal tissue. In contrast, HRT measures the topography of the surface of the retina, with the position of a reference plane 50 μm below the temporal edge of the optic nerve head (ONH) being used to distinguish between cup and rim. These differences lead to RNFL estimates by the two devices that cannot be directly compared. To illustrate this, in Figure 1 the ONH parameters (cup area, cup/disc area ratio) and the mean RNFL thickness values derived from HRT versus OCT, obtained on the same eye for each participant of the Indianapolis Glaucoma Progression Study (IGPS) [57], are plotted. In the IGPS, a longitudinal study that was aimed at evaluating the relationship between ocular hemodynamics and glaucoma progression, 115 OAG patients were assessed every 6 months over a 7 year period for IOP, systolic and diastolic blood pressures (SBP, DBP), heart rate (HR), and structural and hemodynamic evaluations via multiple imaging devices, including OCT, HRT, HRF, and CDI [57,58]. As shown in Figure 1, when the same biomarkers were assessed by HRT and OCT, the two instruments provided consistently different results. Therefore, it is important to highlight that differences among instruments pose a serious challenge for the applicability and generalization of AI models across studies.

**Figure 2.**
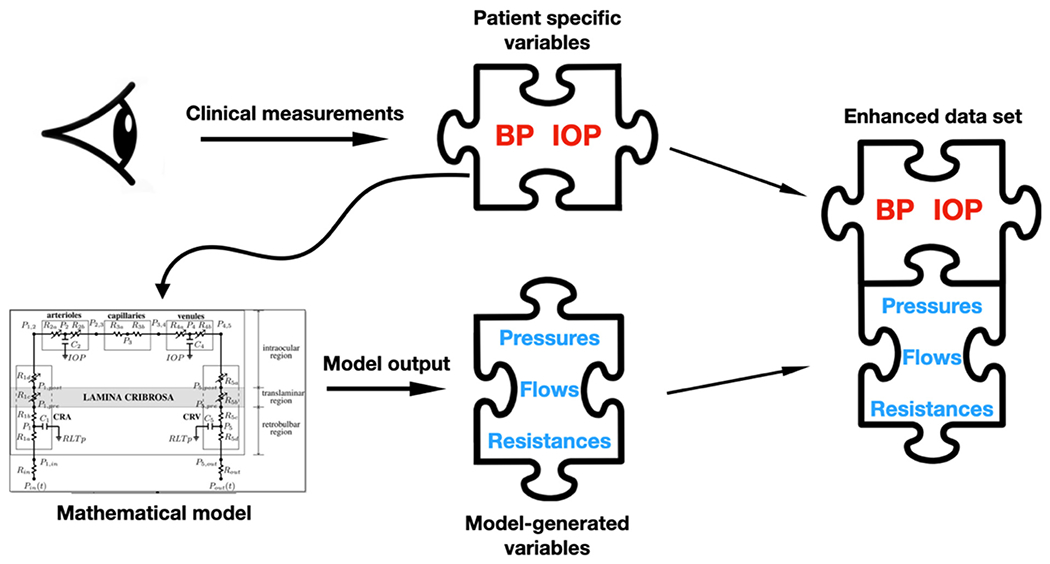
Schematic representation of the data set enhancement process.

In order to overcome these obstacles, a combined approach of AI and principle-based mathematical modeling, called physiology-informed machine learning, was proposed in a series of abstracts presented at the 2022 Annual Meeting of the Association for Vision and Research in Ophthalmology [59–64]. This approach is founded on the observation that rather than focusing on the discrepancies in or unavailability of the data, it is possible to find a unifying framework in the immutable principles of physiology. A mechanistic mathematical model of retinal circulation is used to predict hemodynamic variables that cannot be directly measured. The mathematical model only requires four inputs: IOP, SBP, DBP, and HR, all of which are readily accessible variables available in all glaucoma clinical studies. The variables generated by the model are combined with clinical measurements to create an enhanced dataset (see Figure 2). The idea is that this enhanced dataset carries information obtained from physiological principles that are consistent across studies and populations, and the information is less likely to suffer from the shortcomings of the raw instrument measurements discussed above. More precisely, the mathematical model is capable of capturing complicated (non-linear) interactions between IOP and BP that may produce extreme physiological responses, such as venous collapse (see the discussion on [60], below). AI models may be unable to detect these dynamics from the raw experimental data available, but they are able to do so by including the hemodynamic variables generated by the mathematical model in the datasets.

As a proof of concept, this approach has been recently tested on the IGPS. In [59], the fuzzy C-means algorithm was applied to the enhanced dataset to reveal three clusters of patients (Cluster 1, Cluster 2, and Cluster 3) that were analyzed in terms of their clinical outcomes after four years. It was found that Cluster 1 patients showed minimal progreasion, Cluster 2 patients showed both structural progression and hemodynamics changes, and Cluster 3 patients showed changes only in hemodynamic variables.

In [60], the three clusters were analyzed in terms of their hemodynamic behavior. Ocular perfusion pressure (2/3MAP-IOP) was found to be high in Cluster 2 patients and low in Cluster 3 patients. Moreover, while the median of the peak-systolic velocity (PSV) in the central retinal artery was similar for patients in all three clusters, the PSV in the ophthalmic artery was higher in Cluster 2 patients than for patients in the other clusters. On the other hand, Cluster 3 patients exhibited higher vascular resistance in the venules and the central retinal vein. These results suggested that high and low blood pressure, in combination with IOP, may impact glaucoma through different mechanisms. Specifically, patients in Cluster 2 may need stronger autoregulation engagement to maintain homeostasis, rendering the system unable to compensate for physiological fluctuations in blood pressure. The high vascular resistance seen in Cluster 3 patients may be an indication that those vessels are susceptible to venous collapse.

**Figure 3.**
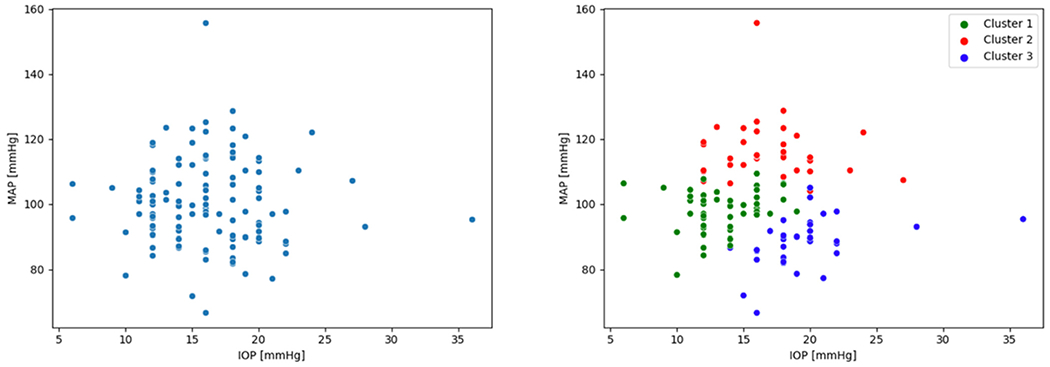
In both graphs, each dot represents a patient in the IGPS, plotted with respect to IOP and mean arterial pressure (MAP). On the right, patients are colored according to the label assigned to them by the fuzzy C-means algorithm applied to the enhanced dataset. The physiology-informed machine learning approach was able to quantify relative contributions of IOP and blood pressure to OAG risk for patients in these clusters.

Attempts to extend this analysis beyond the IGPS have also been made [61–64]. In [61–63], the clusters obtained in the IGPS were transferred via transfer learning to a dataset consisting of 56 patients (11 of which had glaucoma) that was collected at the Mount Sinai School of Medicine and analyzed with respect to various markers, such as optical coherence tomography angiography, oximetry, and choroidal thickness. In [64], the physiology-informed machine learning approach was used to analyze the Thessaloniki Eye Study [65] and the Singapore Epidemiology of Eye Disease Study [52], along with the IGPS. As opposed to the IGPS, [52,65] were large population-based studies containing only a small portion of glaucoma eyes. When applying the same clustering techniques used for studying the IGPS (which contains only glaucoma eyes) to [52,65], no clear patterns emerged. However, a clustering structure such as the one displayed in Figure 3 (right) was found when considering only glaucoma eyes. On one hand, this indicates that relevant patterns might be obscured by a disproportionate presence of healthy eyes in a given dataset. On the other hand, the physiology-informed machine learning approach might be able to reveal structures among glaucomatous eyes that are common to studies across nationalities and ethnicities.

## 7. Conclusions and Perspectives

As AI-assisted screening, diagnosis, and treatment has been gaining momentum within ophthalmology, it is important to understand and address the barriers to clinical adoption. Factors that influence adoption have been well studied, especially in the fields of healthcare delivery and technology. Rogers’ Diffusion of Innovation Theory [66,67] can offer a useful framework for understanding clinical adoption by examining five major attributes of the innovation: (1) relative advantage; (2) compatibility; (3) complexity; (4) trialability; and (5) observability.

Early pilot research [68] examined provider understanding and adoption of AI within the field of ophthalmology using a sample of 18 clinical providers. The results indicated that nuanced barriers exist, particularly a lack of clinical buy-in and a lack of availability of big datasets, as previously discussed. Future research should build upon the noted challenges to develop a rich understanding of the barriers to translating and communicating the science to clinical practice.

As discussed previously, it has been demonstrated that the combining of physics-based models in the modeling of glaucoma has the potential to provide additional information to clinical observations. Combined neural network/physics models are a subject of intensive current research (see, e.g., [69]), and such methods could certainly be applied to mechanistic models in glaucoma research. An alternative approach is to consider so-called physical-statistical models (see, e.g., the overviews in [70,71]) that seek to combine various types of observations, deterministic model output, and physical relationships within a BHM framework. To date, this approach has been applied in many areas of science (e.g., meteorology, climatology, and ecology), but it has not been implemented in the context of glaucoma research. In this context, a related area of potential impact in glaucoma research is the data-driven discovery of physical mechanisms. This approach has recently become an active area of research in the applied mathematics community (see, e.g., [72]) but it has yet to be implemented in the context of glaucoma mechanistic model discovery.

While AI is a formidable tool that can help us discern patterns that are buried in large datasets, its effective use necessarily entails a thorough understanding of its limitations. As discussed in Section 6 and as illustrated in Figure 1, different measuring instruments can yield discrepant readings when attempting to measure the same ocular parameter. Further, even if these discrepancies are not present, it is important to be aware that the choice of data to be collected is heavily influenced by our current theories and perspectives. If a given quantity of information is deemed to be important for understanding certain phenomenon, it is more likely that resources will be allocated to develop measuring instruments for the task, which would in turn render this variable easily accessible and cause it to feature in most collected datasets. AI models trained on this data may overplay the importance of this variable, in detriment of other quantities (for a thorough report on the risk of bias in AI, see [16]). Thus, relying exclusively on the data may result in a circular confirmation of our own biases and hinder the possibility of true scientific discovery.
